# Residual inflammatory risk in coronary heart disease: incidence of elevated high-sensitive CRP in a real-world cohort

**DOI:** 10.1007/s00392-019-01511-0

**Published:** 2019-07-19

**Authors:** Alexander Peikert, Klaus Kaier, Julian Merz, Lucas Manhart, Ibrahim Schäfer, Ingo Hilgendorf, Philipp Hehn, Dennis Wolf, Florian Willecke, Xia Sheng, Andreas Clemens, Manfred Zehender, Constantin von zur Mühlen, Christoph Bode, Andreas Zirlik, Peter Stachon

**Affiliations:** 1 Department of Cardiology and Angiology I, University Heart Center Freiburg, Medical Faculty, University of Freiburg, Freiburg, Germany; 2 Institute of Medical Biometry and Statistics, Faculty of Medicine and Medical Center, University of Freiburg, Freiburg, Germany; 3 Faculty of Biology, University of Freiburg, Freiburg, Germany; 4 Novartis Pharma AG, Basel, Switzerland; 5 Department of Cardiology, Medical University of Graz, Graz, Austria

**Keywords:** hsCRP, Cardiovascular disease, Inflammation, Real-world cohort, Coronary heart disease, LDL-cholesterol

## Abstract

**Background:**

Inflammation drives atherosclerosis and its complications. Anti-inflammatory therapy with interleukin 1 beta (IL-1β) antibody reduces cardiovascular events in patients with elevated high-sensitive C-reactive protein (hsCRP). This study aims to identify the share of patients with coronary heart disease (CHD) and residual inflammation who may benefit from anti-inflammatory therapy.

**Methods:**

hsCRP and low-density lipoprotein (LDL) levels were determined in 2741 all-comers admitted to the cardiological ward of our tertiary referral hospital between June 2016 and June 2018. Patients without CHD, with acute coronary syndrome, chronic or recurrent systemic infection, use of immunosuppressant or anti-inflammatory agents, chronic inflammatory diseases, chemotherapy, terminal organ failure, traumatic injury and pregnancy were excluded.

**Results:**

856 patients with stable CHD were included. 42.7% of those had elevated hsCRP ≥ 2 mg/l. Within the group of patients with LDL-cholesterol < 70 mg/dl, 30.9% shared increased hsCRP indicating residual inflammation. After multivariate adjusted backward selection elevated Lipoprotein (a) (OR 1.61, *p* = 0.048), elevated proBNP (OR 2.57, *p* < 0.0001), smoking (OR 1.70, *p* = 0.022), and obesity (OR 2.28, *p* = 0.007) were associated with elevated hsCRP. In contrast, the use of ezetimibe was associated with normal hsCRP (OR 0.51, *p* = 0.014). In the subgroup of patients with on-target LDL-cholesterol < 70 mg/dl, backward selection identified elevated proBNP (OR 3.49, *p* = 0.007) as independent predictor of elevated hsCRP in patients with LDL-cholesterol < 70 mg/dl.

**Conclusion:**

One-third of all-comers patients with CHD showed increased levels of hsCRP despite a LDL-cholesterol < 70 mg/dl potentially qualifying for an anti-inflammatory therapy. Elevated proBNP is an independent risk factor for hsCRP elevation.

**Graphic abstract:**

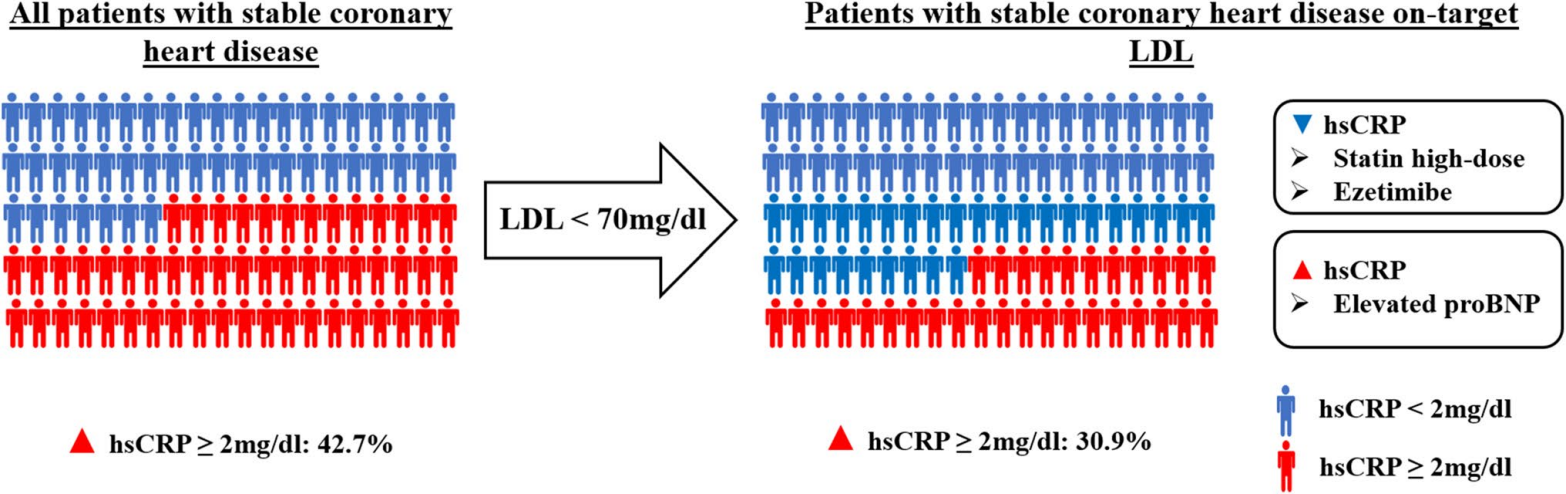

**Electronic supplementary material:**

The online version of this article (10.1007/s00392-019-01511-0) contains supplementary material, which is available to authorized users.

## Introduction

Vast evidence from clinical and experimental studies showed that vascular inflammation is a key process in the development of atherosclerosis [1, 2]. Elevated hsCRP is associated with an increase of cardiovascular events and, therefore, serves as an independent biomarker for cardiovascular risk [3, 4]. Statins are powerful lipid-lowering drugs, but they reduce lipid-independently hsCRP and prevent thereby cardiovascular events [5, 6]. However, experimental data suggest that hsCRP indicates vascular inflammation as a marker, but is not directly involved in the pathogenesis of atherosclerosis [7]. Over more than 2 decades, multiple specific targets for an anti-inflammatory and anti-atherogenic therapy were identified by basic research. However, several randomised controlled clinical trials failed to prove their efficacy in humans [8]. In 2017, the Canakinumab Anti-inflammatory Thrombosis Outcomes Study (CANTOS) showed for the first time improved cardiovascular outcomes in patients with increased hsCRP due to a specific anti-inflammatory therapy independent of lipid-lowering with the IL-1β antibody canakinumab [9, 10]. Therefore, anti-inflammatory therapy may emerge as a new treatment option beside risk factor modulation, platelet inhibition, and lipid lowering for patients suffering from stable CHD. Understanding who benefit from an anti-inflammatory therapy in real world is mandatory to implement this new therapy concept from randomised controlled setting into clinical practice. The present study analyses how many patients with CHD reveal an increased residual inflammatory activity in a real-world cohort. Aim of the study is to define the need for an anti-inflammatory therapy as well as the identification of predictors of residual inflammatory activity in patients with CHD in a real-world setting.

## Methods

### Trial design

This investigator-initiated cross-sectional retrospective cohort study was conducted at the University Heart Center Freiburg. The trial design resonates with the tenets of the revised Helsinki protocol. All study participants provided written informed consent for retrospective data analyses at point of hospital admission before being screened. The recruitment patient collective represents a mid-western population of predominant Caucasoid European ancestry.

Between June 2016 and June 2018, 2741 all comer patients admitted at the Department of Cardiology and Angiology I at the University Heart Center Freiburg, a tertiary hospital, with complete blood profile including hsCRP and LDL-cholesterol were screened for eligibility to be included. During the screening period, the determination of LDL-cholesterol and hsCRP was routine at admission in all patients. Patients without CHD, with acute coronary syndrome, chronic or recurrent systemic infection, use of immunosuppressant or anti-inflammatory agents, chronic inflammatory diseases, chemotherapy, history or risk of tuberculosis, human immunodeficiency virus, terminal organ failure, traumatic injury, or pregnancy were excluded. After completion of the screening period, the included collective was divided into the following groups for cross-sectional retrospective analysis: patients sharing inflammatory activity with hsCRP of 2 mg/l or more, patients without inflammatory activity with hsCRP smaller than 2 mg/l, patients with off target LDL-cholesterol levels 70 mg/dl or more and patients with on-target LDL-cholesterol levels smaller than 70 mg/dl. To define factors influencing hsCRP, univariate logistic regression and backward selection were performed. Laboratory biomarker levels (including hsCRP and LDL-cholesterol) at admission were used for analyses.

### Statistical analysis

In a first step, a descriptive analysis of patients with hsCRP ≥ 2 or < 2 mg/l and patients with LDL-cholesterol levels ≥ 70 and < 70 mg/dl was performed. The impact of different risk factors on hsCRP and LDL was analysed using univariate logistic regression analyses. In the purpose to identify a group of risk factors for risk prediction, a backward selection process was applied. Therefore, a multivariate adjusted backward selection process was applied. Starting with all risk factors in a multivariate logistic regression analysis, in each round, the risk factor whose loss gives the most statistically insignificant deterioration of the model fit is excluded. This process is repeated until a certain significance level for removal from the model is reached (*p* ≤ 0.05). Univariate logistic regression and multivariate backward selection process models both included the variables “female sex”, “age > 75”, “Lipoprotein (a) > 30 mg/dl”, “LDL-Cholesterol > 70 mg/dl”, “HbA1c > 6,5%”, “proBNP > 500 pg/ml”, “hypertension”, “diabetes”, “smoking”, “BMI > 30 kg/m^2^”, “stable CHD with revascularization procedure”, “history of acute coronary syndrome”, “no statin”, “statin low-dose”, “statin high-dose, “ezetimibe”, “PSCK9-inhibitor” and “metabolic syndrome”. Missing variables were addressed by no imputation. Analyses were performed including all, respectively, available data. Stata 15.1 was used as statistical software.

### Definitions

Initially, exclusion criteria were pre-specified to ensure adequate patient allocation. The absence of CHD was defined by nonexistence of the diagnosis regarding patient’s medical records. Stable CHD was defined as CHD confirmed by coronary angiography without presentation of an acute coronary syndrome (comprising STEMI, NSTEMI, unstable angina) during the period of the respective hospital stay. According to the exclusion criteria of the CANTOS trial, patients with contemporary acute coronary syndrome during the screening period were excluded to avoid interference of acute phase immunological activation after recent myocardial ischemia and inflammatory biomarkers. Systemic infection was defined by suspected or proven state of current and/or chronic (including tuberculosis, hepatitis B and hepatitis C) infections of all causes. As a surrogate of potential states of current infections, patients with CRP levels ≥ 15 mg/l were excluded. Patients with immunocompromised states were defined as patients with suspected or proven Human Immunodeficiency Virus (HIV) infection, documented immune deficiency disease and systemic or local treatment with any immune modulating agents (steroids and all other immunosuppressant drugs). Thus, patients with suspected or documented inflammatory bowel diseases, COPD, rheumatologic disorders, auto-immune and auto-inflammatory diseases were defined as chronic inflammatory diseases. To avoid inclusions of patients with terminal organ failure, the defined collective of patients with known active or recurrent hepatic disorder, terminal renal insufficiency and haemodialysis, terminal lung diseases of all origins (including obstructive and restrictive pulmonary disorders) and patients after organ transplantation were excluded. Furthermore, patients with cancer under ongoing chemotherapy independent of cancer type were excluded per definition. Pregnancy was defined as state after conception until termination of gestation including confirmation by a positive hCG laboratory test.

## Results

### Study population

**Fig. 1:**
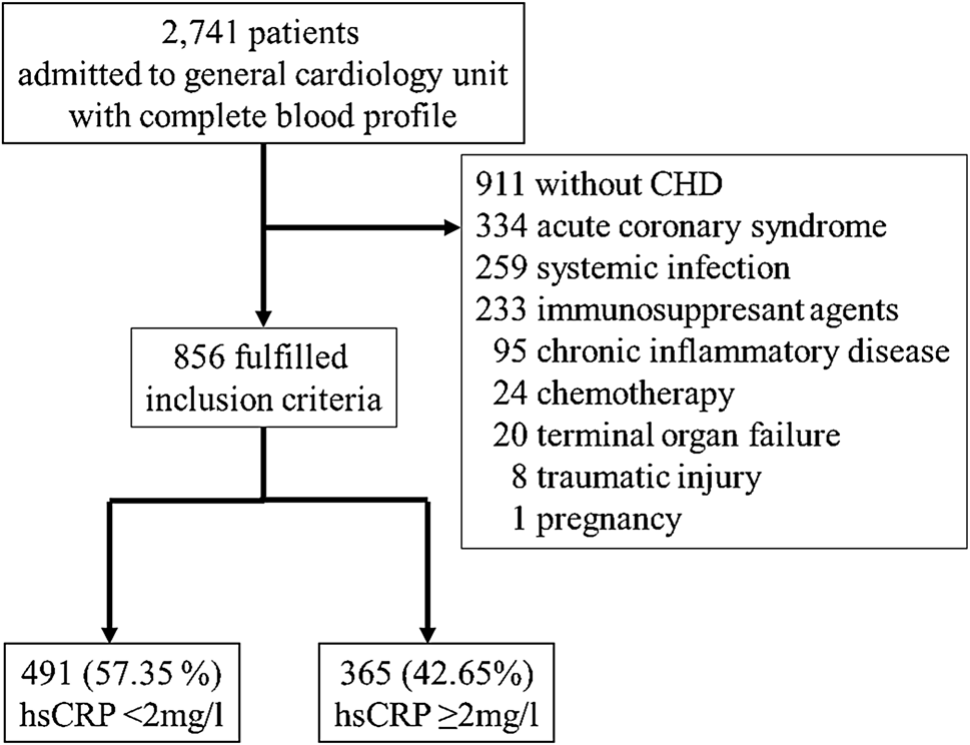
Consort diagram. 2741 patients admitted to general cardiology with complete blood profile were screened. 1885 patients were excluded due to lack of CHD (911), acute coronary syndrome (334), systemic infection (259), use of immunosuppressant agents (233), chronic inflammatory diseases (95), chemotherapy (24), terminal organ failure (20), traumatic injury (8) and pregnancy (1). hsCRP was increased in 42.65% (365) of the patients, 57.35% (491) showed normal hsCRP-values

During the period between June 2016 and June 2018, 2741 patients admitted to general cardiology ward with complete blood profile were screened. 1885 Patients were excluded due to lack of CHD (911), acute coronary syndrome (334), systemic infection (259), chronic inflammatory diseases (95), chemotherapy (24), terminal organ failure (20), traumatic injury (8), use of immunosuppressant agents (233), or pregnancy (1). Finally, 856 patients fulfilled the inclusion criteria (Fig. 1).

### Baseline characteristics

The mean age within the included study population was 71.8 years. The share of females was 27%. The majority (80%) of the study population had hypertension, 29% suffered from diabetes, and 40% were smokers. The average BMI was 26.82. The vast majority of patients were admitted for elective procedures such as coronary angiography, valvular interventions, pacemaker/ICD-device interventions or electrophysiological procedures. Less than half of patients (39%) had undergone revascularisation procedures during the hospital stay, and one-third (34%) had history of acute coronary syndrome. Statins were taken by altogether 76% (26% low dose, 50% high dose) of patients, ezetimibe by 20%, PCSK9 inhibitors by 1% (Table 1). The mean hsCRP was 2.4 mg/l. hsCRP was increased in 42.6% (365) of all patients. Patients with hsCRP ≥ 2 mg/l had increased levels of Lipoprotein (a) (*p* = 0.055), LDL-cholesterol (*p* < 0.001), proBNP (*p* < 0.001), were more often smokers (*p* < 0.001), suffered from diabetes (*p* = 0.034) and had a BMI > 30 kg/m^2^ (*p* < 0.001). The use of statins and ezetimibe was lower in the hsCRP ≥ 2 mg/l group (*p* = 0.009, *p* < 0.001). After backward selection elevated Lipoprotein (a) (OR 1.61, 95% CI 1.00–2.57, *p* = 0.048), elevated proBNP (OR 2.57, 95% CI 1.64–4.02, *p* > 0.001), smoking (OR 1.70, 95% CI 1.08–2.70, *p* = 0.022), and BMI > 30 kg/m^2^ (OR 2.28, 95% CI 1.26–4.12, *p* = 0.007) were independent risk factors for elevated hsCRP. In contrast, medication with ezetimibe was protective (OR 0.51, 95% CI 0.30–0.87, *p* = 0.014, Table 2).

**Table 1:** Baseline characteristics

Characteristic	All patients	hsCRP < 2 mg/l	hsCRP ≥ 2 mg/l
*n*	856	491	365
Female sex	27%	27%	27%
Age (yrs)	71.75	72.08	71.32
hsCRP (mg/l)	2.43	0.91	4.48
Lipoprotein (a) (mg/dl)	33.33	30.78	36.63
LDL-cholesterol (mg/dl)	100.44	95.89	106.30
HbA1c (%)	6.11	6.04	6.19
proBNP (pg/ml)	1456.05	899.18	2294.60
Hypertension	80%	80%	81%
Diabetes	29%	26%	32%
Smoking	40%	35%	48%
BMI (kg/m^2^)	26,82	26,23	27,63
Stable CHD with revascularization procedure	39%	38%	40%
History of acute coronary syndrome	34%	34%	34%
No statin	24%	20%	30%
Statin low dose	26%	26%	25%
Statin high dose	50%	54%	45%
Ezetimibe	20%	24%	14%
PSCK9-inhibitor	1%	1%	1%

Normally distributed continuous variables are expressed as means; categorical variables are expressed as percent counts

*yrs* years, *BMI* body mass index, *CHD* coronary heart disease

**Table 2:** Multivariate analysis of factors influencing hsCRP elevation in all included patient

Characteristic	All patients (*n* = 856)							
			Univariate logistic regression		Multivariate logistic regression analysis with backward selection			
	hsCRP < 2 mg/l (*n* = 491) (%)	hsCRP ≥ 2 mg/l (*n* = 356) (%)	Odds ratio	*p* value	Odds ratio	*p* value	95% CI	
Female sex	27	27	0.97	0.83				
Age > 75 yrs	45	42	0.90	0.43				
Lipoprotein (a) > 30 mg/dl	28	34	1.34	0.05	1.61	0.048	1.00	2.57
LDL-cholesterol > 70 mg/dl	67	79	1.84	< 0.001				
HbA1c > 6.5%	18	22	1.33	0.10				
proBNP > 500 pg/ml	37	60	1.55	< 0.001	2.57	< 0.001	1.64	4.02
Hypertension	80	81	1.06	0.73				
Diabetes	26	32	1.38	0.03				
Smoking	35	48	1.71	< 0.001	1.70	0.022	1.08	2.70
BMI > 30 kg/m^2^	13	26	2.29	< 0.001	2.28	0.007	1.26	4.12
Stable CHD with revascularization procedure	38	40	1.07	0.61				
History of acute coronary syndrome	35	35	0.97	0.81				
No statin	20	30	1.76	< 0.001				
Statin low dose	26	25	0.93	0.64				
Statin high dose	54	45	0.69	0.009				
Ezetimibe	24	14	0.53	0.001	0.51	0.014	0.30	0.87
PSCK9-inhibitor	1	1	1.34	0.72				
Metabolic syndrome	23	5	0.17	< 0.001				

### Cholesterol and hsCRP levels

**Fig. 2:**
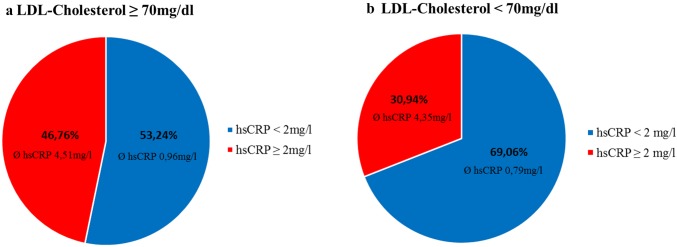
Share of patients regarding LDL-cholesterol levels and hsCRP. In patients with a LDL-cholesterol ≥ 70 mg/dl, 46.76% have increased hsCRP levels (*Ø* 4.51 mg/l) (**a**). 30.94% of patients with a LDL-cholesterol < 70 mg/dl show elevated levels of hsCRP (*Ø* 4.35 mg/l) suggestive of residual inflammation (**b**)

The mean LDL-cholesterol of the study population was 100.4 mg/dl. The LDL target of < 70 mg/dl was reached in 26.1% (*n* = 223) of the patients assuming an optimal treatment of hypercholesterinaemia. Consequently, this group of patients took more frequently statins (LDL < 70 mg/dl 94% vs LDL ≥ 70 mg/dl 69%, Supplemental Table). Patients with on-target cholesterol levels showed significantly lower mean hsCRP concentrations compared to patients with off target cholesterol values (1.89 mg/l vs 2.76 mg/l, *p* = 0.0001). As expected, the share of patients with elevated hsCRP was higher (46.8%) in the group with LDL-cholesterol ≥ 70 mg/dl (LDL ≥ 70 mg/dl, hsCRP > 2 mg/dl: mean hsCRP 4.51 mg/l, LDL ≥ 70 mg/dl, hsCRP < 2 mg/dl: mean hsCRP 0.96 mg/dl). Nevertheless, 30.9% (*n* = 69) of patients with on-target LDL-cholesterol levels still showed elevated levels of hsCRP (LDL < 70 mg/dl, hsCRP > 2 mg/dl: mean hsCRP 4.35 mg/l, LDL < 70 mg/dl, hsCRP < 2 mg/dl: mean hsCRP 0.79 mg/dl, Fig. 2).

### Predictors for elevated hsCRP in patients with LDL < 70 mg/dl

LDL-cholesterol is associated with hsCRP elevation and LDL-cholesterol < 70 mg/dl is recommended for patients at high risk for recurrent cardiovascular events. Therefore, we analysed the residual inflammatory activity defined as hsCRP ≥ 2 mg/l in patients with optimal LDL-cholesterol. Elevated proBNP (OR 3.15, *p* = 0.004), arterial hypertension (OR 2.31, *p* = 0.046), BMI > 30 kg/m^2^ (OR 2.22, *p* = 0.028), and use of low-dose statins (OR 2.37, *p* = 0.006) were associated with increased risk for elevated hsCRP. In contrast, high-dose statins (OR 0.35, *p* = 0.001) and ezetimibe (OR 0.26, *p* < 0.001) decreased the risk for elevated hsCRP. After multivariate backward selection, elevated proBNP was an independent predictor of elevated hsCRP in patients with LDL-cholesterol < 70 mg/dl (OR 3.49, 95% CI 1.41–8.60, *p* = 0.007). In contrast, the use of high-dose statins (OR 0.29, 95% CI 0.11–0.72, *p* = 0.007) or ezetimibe (OR 0.30, 95% CI 0.13–0.96, *p* = 0.042) reduced independently hsCRP levels in patients with target LDL-cholesterol (Table 3).

**Table 3:** Multivariate analysis of factors influencing hsCRP elevation in all patients with LDL < 70 mg/dl

Characteristic	Subgroup LDL < 70 mg/dl (*n* = 223)							
			Univariate logistic regressions		Multivariate logistic regression analysis with backward selection			
	hsCRP < 2 mg/l (*n* = 154) (%)	hsCRP ≥ 2 mg/l (*n* = 69) (%)	Odds ratio	*p* value	Odds ratio	*p* value	95% CI	
Female sex	18	16	0.85	0.658				
Age > 75 yrs	39	46	1.35	0.287				
Lipoprotein (a) > 30 mg/dl	24	32	1.52	0.180				
LDL-cholesterol > 70 mg/dl	0	0	1.00					
HbA1c > 6.5%	22	23	1.01	0.969				
proBNP > 500 pg/ml	35	63	3.15	0.004	3.49	0.007	1.41	8.60
Hypertension	79	89	2.31	0.046				
Diabetes	30	34	1.21	0.515				
Smoking	43	46	1.13	0.664				
BMI > 30 kg/m^2^	13	25	2.22	0.028				
Stable CHD with revascularization procedure	43	39	0.88	0.659				
History of acute coronary syndrome	50	45	0.83	0.506				
No statin	2	8	3.32	0.070				
Statin low dose	19	36	2.37	0.006				
Statin high dose	79	56	0.35	0.001	0.29	0.007	0.11	0.72
Ezetimibe	42	16	0.26	< 0.001	0.35	0.042	0.13	0.96
PSCK9-inhibitor	0	1	1.00					
Metabolic syndrome	24	30	1.35	0.338				

*yrs* years, *BMI* body mass index, *CHD* coronary heart disease

## Discussion

The present study investigated in a real-world all-comers collective the share of patients with CHD, which have a residual inflammation and may, therefore, benefit from an anti-inflammatory therapy. The recently published CANTOS trial demonstrated a reduction of cardiovascular mortality with canakinumab, an IL-1β antibody, in patients at high cardiovascular risk and residual inflammatory activity defined by an elevated hsCRP ≥ 2 mg/l population including patients with a history of myocardial infarction [9]. hsCRP and LDL-cholesterol was determined between June 2016 and June 2018 in 2741 patients, but 856 patients of our population finally fulfilled inclusion criteria of CANTOS regarding a history of CHD. In contrast to the CANTOS trial, we included all patients with known CHD, whereas CANTOS included patients with a myocardial infarction more than 30 days ago [11]. Nevertheless, only patients with stable CHD are candidates for a therapy with canakinumab, since experimental data showed that IL-1β blockage in the acute phase of a myocardial infarction increased the occurrence of ventricular rupture [12]. Therefore, patients with acute coronary syndrome were excluded in CANTOS and consequently in our study. We observed that roughly half of the patients with stable CHD revealed inflammatory activity in daily clinical practice and may, therefore, qualify for an anti-inflammatory treatment such as canakinumab. This confirms the results from the randomised controlled Improved Reduction of Outcomes: Vytorin Efficacy International Trial (IMPROVE-IT), where 47% of patients with previous myocardial infarction revealed increased levels of hsCRP 1 month after index event [13].

In our study, more than 77% did not achieve recent guideline-conform LDL-cholesterol targets of less than 70 mg/dl. 24% did not receive statins despite a known CHD. This finding is in accordance with a large multicentre observation, which found similar cholesterol target value attainments [14, 15]. However, the efficacy of LDL-lowering therapy for patients with CHD is well established for statins and was contemporary proven by the three randomised controlled PCSK9-inhibitor trials [6, 16–19]. According to other clinical studies, LDL-levels < 70 mg/dl were associated with lower hsCRP concentrations underlining the important role of LDL-cholesterol in vascular inflammation [6, 20]. Thus, LDL-cholesterol target is < 70 mg/dl according to the recent guidelines to reduce both, lipid-driven cardiovascular risk and inflammation [21, 22]. Lately, there is upcoming evidence from post hoc analysis of the PCSK9 inhibitor trials, showing that a further reduction of LDL-cholesterol below current guideline targets using PCSK9 inhibition reduced vascular events, but had no effect on hsCRP plasma concentrations [23, 24]. Supported by various previous evidences from basic science, these clinical data suggest a lipid-independent persistence of inflammation independent from LDL-cholesterol. In the present study, about one-third of patients exhibited an elevated hsCRP despite fulfilling the LDL-cholesterol target < 70 mg/dl. In all patients, elevated lipoprotein a, BMI > 30 kg/m^2^, diabetes, smoking, and elevated proBNP > 500 pg/ml were independently associated with residual inflammation. Our data confirm current hypothesis of traditional cardiovascular and metabolic risk factors such as obesity, diabetes and smoking supporting the onset of vascular and adipose inflammation, leading to a state of chronic inflammatory activation accelerating the progress of cardiovascular disease [3, 25]. Supposing an achievement of LDL-cholesterol < 70 mg/dl in clinical practice, we analysed factors, which are associated with elevated hsCRP in the group with LDL-cholesterol < 70 mg/dl. In this group, only proBNP > 500 pg/ml was an independent predictor for residual inflammation. Traditional cardiovascular and metabolic risk factors did not further demonstrate an independent significant predictive validity for residual inflammation in the group of patients achieving LDL-cholesterol levels < 70 mg/dl. Consequently, these findings suggest proBNP > 500 pg/ml as a useful independent predictor for residual inflammatory risk in clinical practise to identify potential patients qualifying for additional anti-inflammatory treatment. The link between inflammation and heart failure is discussed for more than 2 decades [26, 27]. In accordance to our findings, a recent subgroup analysis of the CANTOS trial described improved outcomes in patients with CHD and heart failure after anti-inflammatory-therapy with Canakinumab [28]. In the meantime, there is numerous evidences suggesting that activation of the innate and adaptive immune system plays a critical role in pathogenesis and maintenance of heart failure, various cytokines and chemokines were described as supportive evidence in this content [29, 30]. Both the activation of immunological responses and persisting chronic inflammatory activity are assumed to provoke myocardial remodelling and finally, at least in part, leading to progress of myocardial dysfunction [29]. Whether treatment of heart failure reduces residual inflammation in patients with CHD needs to be further elucidated.

Additional medication of ezetimibe or statins independently reduced the risk for residual inflammation. This effect is likely based on both a distinct LDL-cholesterol reduction by combining statins with ezetimibe and an additional independent anti-inflammatory component as well [13, 31]. Experimental data advise an ezetimibe-mediated inhibition of transendothelial monocyte migration threw regulation of the NF-κB/MAPK pathway in rabbits and mice as one potential mechanism [32, 33]. Yet, additional pleiotropic, anti-inflammatory effects through statin therapy besides cholesterol reduction have already been demonstrated in a large clinical collective [6]. There is compelling experimental evidence suggesting statins to have a broad range of immunomodulatory properties including reduction of cytokine- as well as chemokine-release, modulation of T cell activity and inhibition of leukocyte recruitment threw decreased expression of adhesion molecules [34–36]. Indeed, these findings confirm that medication with statins combined with ezetimibe reduce cholesterol and residual inflammatory risk. Even in patients with LDL-cholesterol < 70 mg/dl, both drugs reduce the risk for residual inflammation.

In line with the current literature, personalised treatments are required looking forward to different groups of patients distinguishing different cardiovascular risk profiles, implicating the need of anti-inflammatory approaches. According to current guidelines, treatment should focus on optimal lipid levels, on lifestyle changes such as dietary changes, weight reduction, physical activity, stopping any exposure to tobacco, and anti-diabetic or anti-hypertensive therapy treatment [37]. These factors are known to reduce cardiovascular events and residual inflammation. Subsequently, patients still showing inflammatory activity despite the mentioned therapeutically implementations, outline a potential target group for anti-inflammatory therapeutically approaches. Within this target group, particularly patients with heart failure have an increased risk of residual inflammation. The lately reported Cardiovascular Inflammation Reduction Trial (CIRT) failed to reduce cardiovascular events as well as levels of interleukin-1β, interleukin-6 and C-reactive protein with low-dose methotrexate, underling the crucial role of targeting the interleukin-1β pathway in the treatment of CHD [38]. Besides targeting the IL-1β pathway with canakinumab, cardiovascular medicine is waiting with great confidence for future results of recently ongoing trails, investigating the effects of further anti-inflammatory agents such as anakinra, and colchicine on cardiovascular event rates [39, 40].

## Limitations

Due to this study’s cross-sectional retroperspective design, certain limitations need to be considered. The non-randomised, single-centre study design implicates a potential for a section bias. This bias was attempted to be minimised by the predefined large-scaled sample size of the study population. Moreover, this study does not provide longitudinal follow-up information. Attributable to the group-specific analytical design, our data do not permit to draw further conclusions considering the distribution of proBNP values. Beyond, our data do not provide additional information about characteristics such as NYHA class, left ventricular ejection fraction (EF) or heart failure treatment. Thus, the specific subgroup of patients with elevated proBNP associated with residual inflammation cannot be characterised subsequently, related to this study’s design. To further address the clinical associations and characteristics of this study’s subgroup analysis, further investigations need to be realised.

## Conclusion

In conclusion, our data elucidate one-third of all-comers patients with CHD sharing residual inflammatory risk, illustrating the need of implicating anti-inflammatory treatments in clinical practice. Moreover, our study suggests elevated proBNP as a useful independent predictor for residual inflammatory risk in clinical practice. Looking forward to a future of personalised cardiovascular precision medicine, subgroup-specific treatment approaches are required in CHD.

## Electronic supplementary material

Below is the link to the electronic supplementary material.

Supplementary material 1 (RTF 105 kb)

## References

[1] Libby P (2002). Inflammation in atherosclerosis. Nature.

[2] Hansson GK (2005). Inflammation, atherosclerosis, and coronary artery disease. N Engl J Med.

[3] Conen D, Ridker PM (2007). Clinical significance of high-sensitivity C-reactive protein in cardiovascular disease. Biomark Med.

[4] Ray KK (2009). Prognostic utility of apoB/AI, total cholesterol/HDL, non-HDL cholesterol, or hs-CRP as predictors of clinical risk in patients receiving statin therapy after acute coronary syndromes: results from PROVE IT-TIMI 22. Arterioscler Thromb Vasc Biol.

[5] Rouleau J (2005). Improved outcome after acute coronary syndromes with an intensive versus standard lipid-lowering regimen: results from the pravastatin or atorvastatin evaluation and infection therapy-thrombolysis in myocardial infarction 22 (PROVE IT-TIMI 22) trial. Am J Med.

[6] Ridker PM (2008). Rosuvastatin to prevent vascular events in men and women with elevated C-reactive protein. N Engl J Med.

[7] Ridker PM (2016). From C-reactive protein to interleukin-6 to interleukin-1: moving upstream to identify novel targets for atheroprotection. Circ Res.

[8] O’Donoghue ML (2014). Effect of darapladib on major coronary events after an acute coronary syndrome: the SOLID-TIMI 52 randomized clinical trial. JAMA.

[9] Ridker PM (2017). Antiinflammatory therapy with canakinumab for atherosclerotic disease. N Engl J Med.

[10] Ridker PM (2017). Relationship of C-reactive protein reduction to cardiovascular event reduction following treatment with canakinumab: a secondary analysis from the CANTOS randomised controlled trial. Lancet.

[11] Ridker PM (2011). Interleukin-1beta inhibition and the prevention of recurrent cardiovascular events: rationale and design of the canakinumab anti-inflammatory thrombosis outcomes study (CANTOS). Am Heart J.

[12] Hwang MW (2001). Neutralization of interleukin-1beta in the acute phase of myocardial infarction promotes the progression of left ventricular remodeling. J Am Coll Cardiol.

[13] Bohula EA (2015). Achievement of dual low-density lipoprotein cholesterol and high-sensitivity C-reactive protein targets more frequent with the addition of ezetimibe to simvastatin and associated with better outcomes in IMPROVE-IT. Circulation.

[14] Gitt AK (2017). Cholesterol target value attainment and lipid-lowering therapy in patients with stable or acute coronary heart disease: results from the dyslipidemia international study II. Atherosclerosis.

[15] Fox KM (2018). Treatment patterns and low-density lipoprotein cholesterol (LDL-C) goal attainment among patients receiving high- or moderate-intensity statins. Clin Res Cardiol.

[16] Trialists CT (2010). Efficacy and safety of more intensive lowering of LDL cholesterol: a meta-analysis of data from 170,000 participants in 26 randomised trials. Lancet.

[17] Robinson JG (2015). Efficacy and safety of alirocumab in reducing lipids and cardiovascular events. N Engl J Med.

[18] Sabatine MS (2017). Evolocumab and Clinical outcomes in patients with cardiovascular disease. N Engl J Med.

[19] Ridker PM (2018). Cardiovascular event reduction with PCSK9 inhibition among 1578 patients with familial hypercholesterolemia: results from the SPIRE randomized trials of bococizumab. J Clin Lipidol.

[20] Nissen SE (2005). Statin therapy, LDL cholesterol, C-reactive protein, and coronary artery disease. N Engl J Med.

[21] Catapano AL (2016). 2016 ESC/EAS guidelines for the management of dyslipidaemias. Eur Heart J.

[22] Soran H, Dent R, Durrington P (2017). Evidence-based goals in LDL-C reduction. Clin Res Cardiol.

[23] Pradhan AD (2018). Residual inflammatory risk on treatment with PCSK9 inhibition and statin therapy. Circulation.

[24] Bohula EA (2018). Inflammatory and cholesterol risk in the FOURIER trial (further cardiovascular outcomes research with PCSK9 inhibition in patients with elevated risk). Circulation.

[25] McEvoy JW (2015). Relationship of cigarette smoking with inflammation and subclinical vascular disease: the multi-ethnic study of atherosclerosis. Arterioscler Thromb Vasc Biol.

[26] Levine B (1990). Elevated circulating levels of tumor necrosis factor in severe chronic heart failure. N Engl J Med.

[27] Kang S (2017). Relationship of high-sensitivity C-reactive protein concentrations and systolic heart failure. Curr Vasc Pharmacol.

[28] Trankle CR (2018). Usefulness of canakinumab to improve exercise capacity in patients with long-term systolic heart failure and elevated C-reactive protein. Am J Cardiol.

[29] Mann DL (2015). Innate immunity and the failing heart: the cytokine hypothesis revisited. Circ Res.

[30] Radenovic S (2018). Systemic inflammation and functional capacity in elderly heart failure patients. Clin Res Cardiol.

[31] Ishibashi T, Takeishi Y (2011). Ezetimibe and vascular inflammation. Curr Vasc Pharmacol.

[32] Qin L (2014). Anti-inflammatory activity of ezetimibe by regulating NF-κB/MAPK pathway in THP-1 macrophages. Pharmacology.

[33] Gómez-Garre D (2009). Ezetimibe reduces plaque inflammation in a rabbit model of atherosclerosis and inhibits monocyte migration in addition to its lipid-lowering effect. Br J Pharmacol.

[34] Jain MK, Ridker PM (2005). Anti-inflammatory effects of statins: clinical evidence and basic mechanisms. Nat Rev Drug Discov.

[35] Jougasaki M (2010). Statins suppress interleukin-6-induced monocyte chemo-attractant protein-1 by inhibiting Janus kinase/signal transducers and activators of transcription pathways in human vascular endothelial cells. Br J Pharmacol.

[36] Mausner-Fainberg K (2008). The effect of HMG-CoA reductase inhibitors on naturally occurring CD4 + CD25 + T cells. Atherosclerosis.

[37] Estruch R (2010). Anti-inflammatory effects of the Mediterranean diet: the experience of the PREDIMED study. Proc Nutr Soc.

[38] Ridker PM (2018). Low-dose methotrexate for the prevention of atherosclerotic events. N Engl J Med.

[39] Morton AC (2015). The effect of interleukin-1 receptor antagonist therapy on markers of inflammation in non-ST elevation acute coronary syndromes: the MRC-ILA heart study. Eur Heart J.

[40] Nidorf SM (2013). Low-dose colchicine for secondary prevention of cardiovascular disease. J Am Coll Cardiol.

