# Supplementary material for: Residual inflammatory risk in coronary heart disease: incidence of elevated high-sensitive CRP in a real-world cohort
Source: Clin Res Cardiol. 2019 Jul 19;109(3):315–23. doi: 10.1007/s00392-019-01511-0 (PMC7042185; doi:10.1007/s00392-019-01511-0)
Supplement: Supplementary file 1 — Supplementary material 1 (RTF 105 kb) [file 392_2019_1511_MOESM1_ESM.rtf]

Supplemental Table. Baseline characteristics groups patients with LDL≥70mg/dl and LDL<70mg/dl
Characteristic	All patients	LDL ≥ 70mg/dl	LDL < 70mg/dl	
n	856	633	223	
Female sex	27%	31%	16%	
Age (yrs)	71.75	72.03	70.96	
hsCRP (mg/l)	2.43	2.62	1.90	
Lipoprotein (a) (mg/dl)	33.33	35.50	27.02	
LDL-Cholesterol (mg/dl)	100.44	115.87	56.24	
HbA1c (%)	6.11	6.09	6.16	
proBNP (pg/ml)	1456.05	1661.61	923.34	
Hypertension	80%	80%	81%	
Diabetes 	29%	27%	32%	
Smoking	40%	39%	43%	
BMI (kg/m2)	26,82	26.88	26.64	
Stable CHD with revascularization procedure	39%	38%	41%	
History of acute coronary syndrome	34%	29%	47%	
No statin	24%	31%	6%	
Statin low-dose	26%	26%	25%	
Statin high-dose	50%	43%	69%	
Ezetimibe	20%	15%	33%	
PSCK9-Inhibitor	1%	1%	0%	

Normally distributed continuous variables are expressed as means; categorical variables are expressed as percent counts.
Abbreviations: yrs, years; BMI, body-mass index; CHD, coronary heart disease.
